# Temsirolimus in overtreated metastatic renal cancer with subsequent use of sunitinib: A case report

**DOI:** 10.3892/ol.2013.1184

**Published:** 2013-02-07

**Authors:** JOSÉ MIGUEL JURADO, IRENE ZARCOS, MAYTE DELGADO, ISABEL BLANCAS, MARTA LEGERÉN, JOSÉ LUIS GARCÍA-PUCHE

**Affiliations:** Oncology Department, Hospital Clínico Universitario San Cecilio, Avenida, Granada 18012, Spain

**Keywords:** temsirolimus, mTOR inhibitors, metastatic renal carcinoma

## Abstract

During the last decade, we have been developing new therapeutic strategies for the treatment of renal cancer, based on knowledge derived from molecular biology. We report a case of long-term renal metastatic cancer progression despite therapy with sunitinib and interleukin, which are the most active drugs in renal cancer. Disease stabilization for 58 weeks was achieved upon sequential use of temsirolimus, following the occurrence of disease progression during angiogenic therapy. The patient demonstrated excellent tolerance without marked symptoms for 10 months. Hypothyroidism and mumps-related adverse events were present. The survival time from diagnosis to lung metastasis was 8 years. Thus, this case demonstrates promising therapeutic effects of the sequential use of tyrosine kinase inhibitors (TKIs) and mammalian target of rapamycin (mTOR) inhibitors during different stages of the disease.

## Introduction

Renal cell cancer accounts for ∼3% of adult malignancies. Approximately one-third of patients exhibit metastatic disease at the time of diagnosis. According to results from Motzer *et al*, the overall median survival time for advanced cases was 10 months and only 45% of patients who had a good prognosis survived for a median follow-up time of two years (1). The rates of response to chemotherapy and hormonotherapy are low (∼10%). For the past 20 years, cytokines have been the main treatment for metastatic renal cell cancer. Therefore, new methods which utilize molecular-targeted therapies, including anti-angiogenic drugs (2) and mammalian target of rapamycin (mTOR) inhibitors, for example everolimus and temsirolimus (3), have emerged. Temsirolimus was approved by the FDA in March 2008*,* based on a phase III randomized trial for advanced renal cell carcinoma that demonstrated a statistically significant improvement in overall survival; the median overall survival was 10.9 months in temsirolimus-treated patients compared with 7.3 months in interferon (IFN)-treated patients (4). Based on these results, patients with a poor prognosis should receive temsirolimus as a first-line treatment. Recommendations for second or successive treatment lines are not currently provided by the main practice guidelines. Following clinical progression, patients who had previously been treated with a vascular endothelial growth factor-targeted agent may benefit from a change of therapy to mTOR inhibitors (5).

## Case report

### Clinical presentation and diagnosis

A 57-year-old male with no relevant pre-existing medical conditions was admitted to the Hospital Universitario Clínico San Cecilio, Avenida, Granada, Spain in February 2000, due to an episode of macroscopic hematuria. The physical examination of the patient at admission was normal. The patient underwent a thyroid function test and this was also normal; the serum thyroid stimulation hormone (TSH) level was 3.9 IU/ml (lower and uppet limit, 0.27–4.20). Routine laboratory data revealed no abnormal findings. During the evaluation, an abdominopelvic computed tomography (CT) scan revealed a solid mass at the inferior pole of the left kidney. A left nephrectomy was performed and the pathology study reported a stage III (pT4N0M0) G2 clear cell carcinoma and a papillary carcinoma involving either the renal pelvis or the extracapsular region.

The study was approved by the Ethics Committee of the Hospital Universitario Cliínico San Cecilio, Granada, Spain. Written informed consent was obtained from the patient.

### Treatment and clinical course

**Figure 1:**
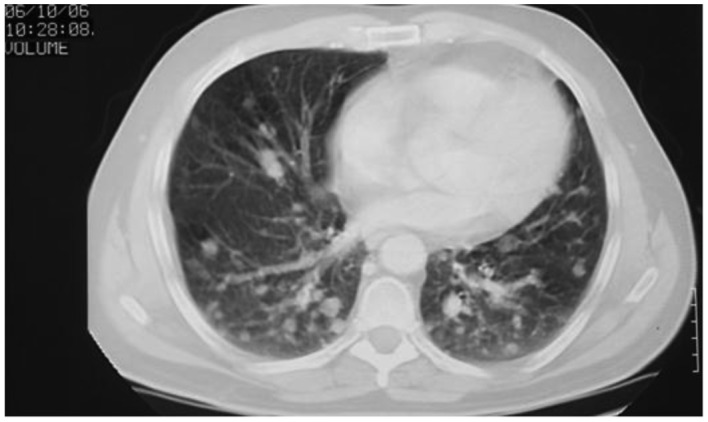
October 2006: The CT scan showed an increase in the number and size of bilateral multiple lung metastases.

**Figure 2:**
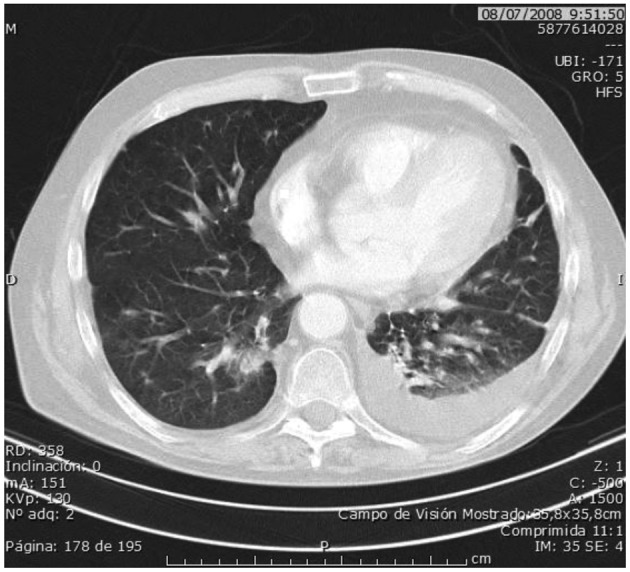
July 2008: The CT scan revealed new pulmonary nodules and pleural effusion.

**Figure 3:**
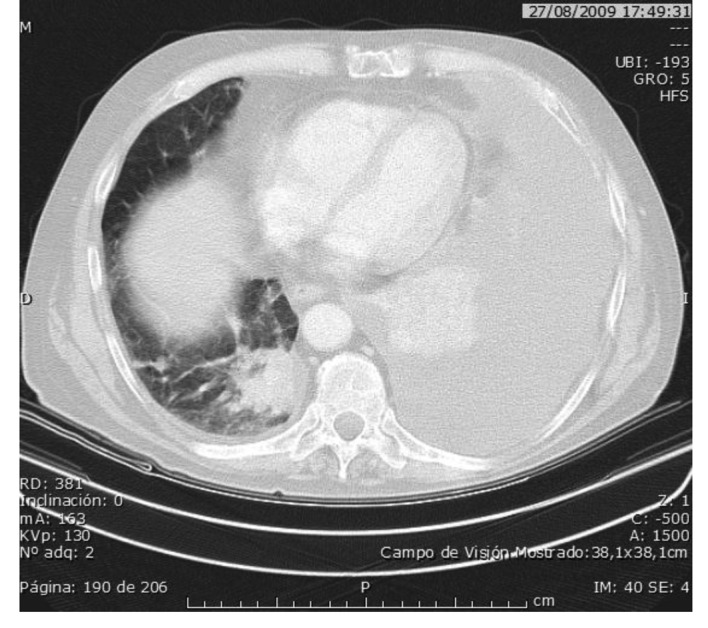
August 2009: The CT scan showed a growth of lung metastases and massive pleural effusion in the left hemithorax.

The patient received adjuvant radiotherapy in the left renal fossa during the phase II trial. Two years later, a CT scan revealed multiple predominant lower lobe metastases. The largest tumor was 2 cm in size and located in the lower left lobe. In December 2002, a first-line treatment with three cycles of intravenous interleukin plus IFN was started; however, there was no response to the therapy. Disease stabilization was achieved following the initiation of cycles of vinblastine and IFN. After 12 cycles of this combination treatment, IFN was administered as a monotherapy. In June 2005, novel progression of the disease to the lungs was revealed. Subsequently, the patient received inhaled interleukin therapy, resulting in the stabilization of the disease over the following 16 months. In October 2006, the CT scan demonstrated an increase in the number and size of bilateral multiple lung metastases (Fig. 1). Fourth-line treatment with sunitinib was subsequently started, and this achieved a long-term partial response over the next 20 months. In July 2008, a new lung relapse was detected (Fig. 2), accompanied by three poor prognostic factors (a low hemoglobin level of <10 g/dl, a serum calcium level of >10 mg/dl and a lactate dehydrogenase level of 709 IU/l). Fifth-line treatment for metastatic disease with 25 mg intravenous temsirolimus once a week was started and disease stabilization was achieved in 13 months. During this period, temsirolimus was discontinued on two occasions. The first incident was due to toxicity (hypothyroidism, G3), which occurred after 10 months of treatment. Severe asthenia and lethargy interfered with activities of daily living in the patient and the results of blood tests were: TSH, 92 *μ*IU/ml; free serum triiodothyroxine (FT3), 0.15 pg/ml (2.60–5.10); and free serum thyroxine (FT4), 0.26 ng/dl (1.00–1.80). The patient achieved normal thyroid function after temsirolimus therapy had been withdrawn for three weeks. In the second instance, the treatment was ceased due to the occurence of mumps accompanied by a high fever; this was treated with antibiotics and anti-inflammatory drugs. Two weeks later, temsirolimus therapy was resumed by lowering the dosage to 20 mg. Notably, other toxicities were observed in this case, including a G2 rash, G2 anemia, G2 leukopenia, G2 hypertriglyceridemia and G1 hypercholesterolemia. In August 2009, the lung disease progression was diagnosed as a massive pleural effusion and treatment was discontinued following evacuation by thoracentesis (Fig. 3).

In September 2009, the patient began a sixth line of treatment with sorafenib and achieved a stable condition for 14 months. However, the patient had a new episode of severe pleural effusion in December 2010 and succumbed to the disease 96 months after the diagnosis of metastases.

## Discussion

Temsirolimus was internally approved at our center, the Hospital Clínico San Cecilio, Avenida, Granada, Spain, in the context of a compassionate use program based on published efficacy data from phase II-III trials (3,6). Before this, randomized trials to support the use of temsirolimus after failure of sunitinib in metastatic renal cancer had not been reported. Atkins *et al*(6) demonstrated the efficacy of temsirolimus treatment in 61% of patients who received temsirolimus therapy as a third-line or later treatment, including in cases where disease progression had occurred following IFN, interleukin or chemotherapy schedules. A report by Gerullis *et al*(7) described a retrospective study in which sunitinib and temsirolimus were sequentially administered for 29 and 6 weeks, respectively. In the present case, the durations of the treatment with sunitinib and temsirolimus were 85 weeks and 58 weeks, respectively.

In a study by Lamm *et al*(8), the authors showed preliminary data with regard to the use of temsirolimus administered in pretreated patients, whose median time to progression was 20 weeks. The results were similar to another study that reported the efficacy achieved with everolimus (9), following the occurrence of disease progression during treatment with sunitinib, sorafenib or both, where 63% disease stabilization and a four-month median time to progression was achieved. Mackenzie *et al* reported the results of 87 patients who had previously been treated with anti-angiogenic therapy; the median time to progression was 4 months and the median overall survival was 11 months (10).

In new-age directed therapies, questions are continuously arising concerning the most effective sequence of drug therapies for increased survival in metastatic renal cell cancer, plus questions with regard to the best methods for identifying accurate predictive markers of clinical efficacy and toxicity (5,11). For second-line treatment, phase III results from the INTORSECT trial on temsirolimus versus sorafenib supports the sequence of tyrosine kinase inhibitor (TKI)-TKI rather than TKI-mTOR (12).

In the current study, following observation of disease progression during sunitinib therapy, we selected to initiate temsirolimus therapy after three poor prognostic factors were exhibited by the patient. Disease stabilization was achieved for one year, with quality-adjusted survival without symptoms for 10 months and the occurrence of two G3 adverse events during the last 3 months of treatment. The common G3 or G4 side-effects with temsirolimus consisted of anemia, hyperglycemia and fatigue/asthenia (13). No cases of hypothyroidism have been reported from the Global ARCC trial. Hypothyroidism is a class-type toxic effect of sunitinib and this event is not associated with temsirolimus. Although the exact pathophysiology of several of these off-target side-effects remains to be determined, it may be explained by inhibition of the same signaling pathways, however at different points (14).

Furthermore, inhibition of the mTOR pathway was accompanied by an increase in cholesterol (326 mg/dl) and triglyceride (426 mg/dl) levels, which may represent a set of markers to indicate drug response efficacy (6). Based upon the rapid progression of renal cancer, we consider this to be a noteworthy case report of metastatic renal cancer in a 57-year-old male. The survival time from diagnosis to lung metastasis was 8 years.

In conclusion, this case report suggests that temsirolimus has significant activity in recurrent renal carcinoma which had been previously treated with interleukin and sunitinib and shows promising effects with regard to the subsequent use of TKI-mTOR-TKI. In the future, biomarkers may allow us to individualize second-line treatments.
